# Magnesium-mediated stress adaptation in plants: from physio-biochemical insights to climate-resilient agriculture

**DOI:** 10.3389/fpls.2026.1715501

**Published:** 2026-02-24

**Authors:** Mohammad Sarraf, Ruchi Bansal, A. M. Shackira, Vaishali Yadav, Saeedeh Zarbakhsh, Rajib Roychowdhury, Devendra Kumar Chauhan, Hesam Mousavi, Mirza Hasanuzzaman

**Affiliations:** 1 Department of Horticultural Science, Faculty of Agriculture, Shahid Chamran University of Ahvaz, Ahvaz, Iran; 2 Division of Plant Physiology, ICAR-Indian Agricultural Research Institute, New Delhi, India; 3 Department of Botany, Sir Syed College, Kannur University, Kerala, India; 4 Department of Botany, Dr. Harisingh Gour Vishwavidyalaya (A Central University), Sagar, Madhya Pradesh, India; 5 Department of Horticultural Science, Faculty of Agriculture, Shiraz University, Shiraz, Iran; 6 International Crops Research Institute for the Semi-Arid Tropics (ICRISAT), Hyderabad, Telangana, India; 7 D D Pant Interdisciplinary Research Laboratory, Department of Botany, University of Allahabad, Prayagraj, India; 8 Faculty of Applied Ecology, Agricultural Science, and Biotechnology, University of Inland Norway, Elverum, Norway; 9 Department of Agronomy, Faculty of Agriculture, Sher-e-Bangla Agricultural University, Sher-e-Bangla Nagar, Dhaka, Bangladesh

**Keywords:** plant nutrition, abiotic stress, plant stress tolerance, photosynthesis, crop productivity

## Abstract

Magnesium (Mg) is a vital macronutrient that underpins multiple processes essential for plant growth, development, and survival. As the central atom in chlorophyll, Mg is indispensable for photosynthesis, the foundation of crop productivity. Beyond light capture, Mg functions as a structural, enzymatic, and regulatory ion, making it a critical mediator of plant tolerance to abiotic stresses. Drought, salinity, extreme temperatures, and nutrient deficiencies continue to limit agricultural yields, yet Mg-mediated pathways can significantly mitigate their effects. By influencing photosynthesis, ion homeostasis, osmotic adjustment, antioxidative defenses, and signal transduction, Mg reinforces multiple layers of plant stress adaptation. This review consolidates current knowledge of Mg’s roles in enhancing plant tolerance to adverse conditions, with particular emphasis on the molecular, physiological, and biochemical mechanisms underlying these roles. By integrating findings across different scales, it advances understanding of Mg-mediated stress adaptation and highlights its potential as a key factor in developing climate-resilient crop production systems. Unlike earlier works that have focused narrowly on Mg nutrition and photosynthesis, this review offers a holistic framework linking molecular insights to agronomic applications. Additionally, it provides future perspectives and research directions to bridge current knowledge gaps and guide innovation in crop breeding, nutrient management, and sustainable production systems.

## Graphical Abstract

**Figure:**
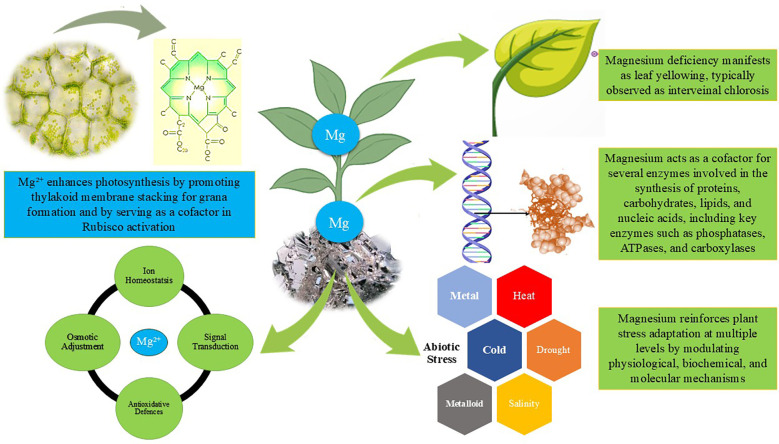

## 1 Introduction

Magnesium (Mg) is an essential macronutrient that significantly impacts plant growth, development, and stress resilience. It plays a key role in numerous physiological and biochemical processes and actively helps plants defend against abiotic stress (Cakmak and White, 2020). As the central atom in the chlorophyll (chl) molecule, Mg is vital for photosynthesis, facilitating light absorption and driving carbon fixation by regulating the activity of RuBP carboxylase in chloroplasts (Gransee and Führs, 2012; Ishfaq et al., 2022). Furthermore, Mg functions as a cofactor for several enzymes involved in the synthesis of proteins, carbohydrates, lipids, and nucleic acids, including ATPases, kinases, phosphatases, Rubisco, and acetyl-CoA carboxylase (Chen et al., 2018). Within mitochondria, these Mg-dependent enzymes are essential for energy production and critical metabolic pathways such as the tricarboxylic acid (TCA) cycle, oxidative phosphorylation, and fatty acid biosynthesis (Chen et al., 2018; Ishfaq et al., 2022).

Magnesium also influences enzymes like nitrate reductase and glutamine synthetase, which are vital for nitrogen assimilation, sugar metabolism, and starch production (Zhang et al., 2023). It is essential for amino acid biosynthesis, aiding both the initiation and elongation of polypeptide chains during protein synthesis, and is key to preserving the structural and functional integrity of ribosomes (Tian et al., 2021). Additionally, Mg forms part of metalloproteins involved in signal transduction pathways that coordinate cellular responses to environmental stimuli (Zhang et al., 2023). However, Mg deficiency is an escalating global issue, especially in highly weathered or calcareous soils, threatening crop yields and food security (Cakmak and White, 2020).

Plants are constantly exposed to various biotic and abiotic challenges that can limit their growth, productivity, and survival (Hassan et al., 2019; Mousavi et al., 2022). Abiotic stress involves harmful non-living factors such as drought, salinity, extreme temperatures, flooding, metal and metalloid toxicity, nutrient deficiencies or imbalances, radiation, and UV-B exposure—further worsened by ozone depletion (He et al., 2018; Shomali et al., 2022). These stresses disrupt physiological, biochemical, and metabolic processes, cause oxidative stress, and result in the excessive build-up of reactive oxygen species (ROS), including hydrogen peroxide (H_2_O_2_), superoxide anion (O_2_•^-^), hydroxyl radical (•OH), and singlet oxygen (¹O_2_) (Sachdev et al., 2021; Sarraf et al., 2024). As a result, oxidative stress hampers the synthesis of DNA, proteins, and carbohydrates, thereby damaging cell walls and organelles (Xie et al., 2019; Noori et al., 2024).

Although plants cannot escape environmental stress, they have developed complex morphological, physiological, and biochemical adaptations (He et al., 2018; Sarraf et al., 2022), including changes in gene expression, hormone signaling, and the synthesis of protective compounds. Antioxidant enzymes such as catalase (CAT), ascorbate peroxidase (APX), superoxide dismutase (SOD), peroxidase (POD), and glutathione reductase (GR), together with non-enzymatic antioxidants like glutathione, ascorbate, phenolics, and carotenoids, play crucial roles in reducing oxidative damage (Sachdev et al., 2021; Johnson et al., 2022). Additional defense mechanisms include restricted translocation of metals, contaminant uptake regulation, root exudation, metal chelation, and the induction of stress-related proteins such as heat shock proteins (HSPs), late embryogenesis abundant (LEA) proteins, dehydrins, and metallothioneins (Yan et al., 2020; Shomali et al., 2024).

Environmental changes that disrupt nutrient and water uptake further impair metabolic processes, growth, and cellular homeostasis (Barzana et al., 2020). Beyond their role as essential macronutrients, minerals are fundamental to cellular organelles and biomolecules—including DNA, RNA, proteins, lipids, and carbohydrates—and participate in crucial metabolic processes such as photosynthesis, respiration, protein and nucleic acid synthesis, and antioxidant metabolism (Singhal et al., 2023).

Given the importance of Mg in plant growth and metabolism, this review consolidates current knowledge of its role in enhancing plant tolerance to abiotic stresses. Notably, the review examines the molecular, physiological, and biochemical mechanisms through which Mg modulates photosynthesis, metabolic regulation, and cellular signaling under adverse conditions. In particular, it highlights how Mg is utilized in physiological reactions, chl biosynthesis, and ion homeostasis to mediate stress adaptation. By integrating findings from recent studies, the review aims to advance understanding of Mg-mediated stress adaptation and shed light on its potential as a key factor in developing climate-resilient crop production systems. Furthermore, this review addresses a critical gap in the literature, as there are few comprehensive reviews of Mg compared to other major nutrients, making it a novel and timely synthesis of Mg’s complex roles in plant physiology.

## 2 Improving magnesium availability in the rhizosphere

### 2.1 Strategies to enhance magnesium availability in the soil

Soil Mg levels generally range from 0.05 to 0.5%, although much of this is readily available to plants. The form accessible to plants is the exchangeable Mg in the soil solution. An optimal Mg concentration of about 120 mg kg^-^¹ is considered beneficial for plant growth and crop yield (Wang et al., 2020; Ishfaq et al., 2022). Regarding dry matter accumulation, the ideal available Mg level falls between 0.07-0.21% in monocots and 0.10-0.70% in dicots (Hauer-Jákli and Tränkner, 2019).

Enhancing Mg levels in plants can be achieved through various sustainable strategies, including agronomic and genetic biofortification. Agronomic biofortification involves increasing Mg availability by applying it directly to the soil, where plant roots take it up via active and passive transport through Mg^2+^ transporters and channels. However, genetic biofortification develops crop varieties with inherently higher Mg content or utilizes genetic modification to improve tolerance to low soil Mg levels (Guo, 2017). Magnesium ions also aid stress tolerance, particularly against abiotic stressors, by competing with other cations such as aluminum (Al), which can trigger antioxidant defenses and modulate the expression of stress-responsive genes, including those encoding HSPs, SOD, CAT, LEA proteins, and dehydrins (Boaretto et al., 2020; Tian et al., 2021).

### 2.2 Impact of soil pH and other factors on magnesium availability

Magnesium in the soil exists in four primary forms: rapidly exchangeable, acid-soluble, organically complex, and structural. Of these, the exchangeable form is readily bioavailable to plants from the soil solution (Mayland and Wilkinson, 1989).

Acidic soils have low Mg availability, and crops grown in such soils often exhibit Mg deficiency due to low cation exchange capacity (CEC, Gransee and Führs, 2012; Chaudhry et al., 2021). However, this is a complex process, and soil pH plays a vital role in regulating Mg bioavailability. Generally, slightly acidic conditions increase the concentration of exchangeable Mg, making it more accessible to plant roots. Nonetheless, excessive H^+^ accumulation in the rhizosphere can hinder Mg uptake, leading to deficiency (Senbayram et al., 2015).

Soils particularly prone to Mg depletion include strongly acidic, highly weathered tropical and subtropical soils, where intense rainfall promotes leaching of exchangeable Mg. These regions frequently experience widespread Mg deficiency due to both natural soil properties and climatic conditions conducive to nutrient loss. Globally, Mg deficiency is increasingly recognized as a major constraint in crop production. For instance, approximately 55% of agricultural soils in China are classified as Mg-deficient (Ishfaq et al., 2022). Similar patterns of Mg insufficiency have been reported in many agroecosystems across Asia, Africa, and Latin America, reflecting the widespread nature of this nutrient limitation.

Magnesium availability is also affected by interactions with other ions, such as calcium and bicarbonates in calcareous soils, as well as Mg carbonates and gypsum in alkaline soils (Farhat et al., 2015; Chaudhry et al., 2021). Additional environmental factors, including temperature, light, and the presence of antagonistic ions like Al, further influence Mg homeostasis (Senbayram et al., 2015). Overall, soil Mg availability depends on its total content, pH, water content, and cation exchange capacity (Wang et al., 2020; Ishfaq et al., 2022).

## 3 Role of magnesium as a plant nutrient

Magnesium serves as a cofactor for metabolic and photosynthetic carbon-fixation enzymes (Marschner, 2012). In fact, 15-35% of the total Mg in mature leaves is associated with the chloroplast (Chen et al., 2018), whereas the remaining Mg is either free as ions or bonded to enzymes, ribosomes, and other functional structures (Marschner, 2012). Magnesium exists in cells as a divalent cation, enabling it to bind electrostatically with nucleophilic ligands. Additionally, Mg regulates the cation-anion balance within cells and, along with K, functions as an osmotically active ion that controls cell turgor (Guo et al., 2016). Therefore, Mg deficiency results in notable impairments in crop growth and yield.

The emergence of Mg deficiency symptoms varies across species, plant parts, and developmental stages. It is suggested that crop plants need about 0.7 mg g^-1^ Mg to reach 90% of their maximum yield (Smith et al., 1985). Since Mg is highly mobile in the phloem, deficiency symptoms often first show on older leaves. A common sign of Mg deficiency is leaf yellowing, which appears as interveinal chlorosis. Typically, chlorosis begins at the tips of mature leaves and spreads along the veins (Cakmak and Kirkby, 2008; Ye et al., 2019). Eventually, anthocyanin pigmentation and necrosis develop between the veins alongside the chlorosis. Chlorosis can further reduce light absorption and CO_2_ fixation. Inhibiting photosynthetic CO_2_ fixation leads to an over-reduction of the photosynthetic electron transport chain and increased ROS production. Therefore, high light intensity during Mg deficiency may accelerate chlorosis and photo-oxidative damage in plants (Mengutay et al., 2013).

During the early stages of Mg deficiency, phloem export of sucrose is severely impaired, often occurring before any visible changes in shoot growth. This disruption leads to a notable increase in the shoot-to-root dry weight ratio, driven by substantial carbohydrate accumulation in source leaves (Chen et al., 2018). Magnesium deficiency severely affects dry matter partitioning between shoots and roots, resulting in significant reductions in yield and crop quality (Grzebisz, 2013; Zhang et al., 2020a). Additionally, Mg is essential for ribosome assembly during protein synthesis; its deficiency causes the accumulation of low-molecular-weight nitrogen compounds, further impairing plant metabolic processes (Yamamoto et al., 2009).

Nonetheless, high Mg concentrations can cause nutrient antagonism, primarily by reducing Ca and K uptake, leading to physiological issues such as leaf chlorosis and poor root growth (Xie et al., 2021). Excess Mg may also disturb osmotic balance and enzyme function, hindering growth. Plants respond by decreasing Mg transporter activity and activating detoxification processes, including vacuolar sequestration and organic acid exudation to preserve ionic balance.

## 4 Magnesium and photosynthesis

### 4.1 Role of magnesium in chlorophyll biosynthesis

Chlorophylls are vital pigment molecules that capture light energy during photosynthesis. The central Mg atom in the tetrapyrrole ring provides the specific absorbance properties needed for energy conversion in the photosynthetic process. The insertion of Mg into protoporphyrin IX marks the initial step in chlorophyll biosynthesis, forming the so-called “Mg branch” in the pathway. This branch point occurs at a crucial stage of metal ion chelation, which is vital for the proper formation and function of the chl molecule. Magnesium^2+^-chelatase is one of the enzymes that catalyses metal-ion insertion. This heterologous enzyme comprises the ChlI (40 kDa), Chld (70 kDa), and ChlH (110 kDa) subunits (Bryant et al., 2020).

Chelation of Mg to the porphyrin ring occurs in two stages (Masuda, 2008). In the first stage, subunits I and D are activated to form a complex with ATP and Mg^2+^, while subunit H binds to protoporphyrin IX and Mg^2+^. During the second stage, Mg chelates into protoporphyrin IX coupled with ATP hydrolysis. Chelation of one Mg requires hydrolysis of 15 ATP (Reid and Hunter, 2004). Magnesium-chelatase activity depends on free Mg concentration. In faba beans, a reduction in Mg-chelatase-submitted H transcripts was reported during early Mg deficiency (Neuhaus et al., 2013).

### 4.2 Magnesium in photosynthetic and metabolic enzymes

#### 4.2.1 Magnesium as an enzyme cofactor

**Figure 1:**
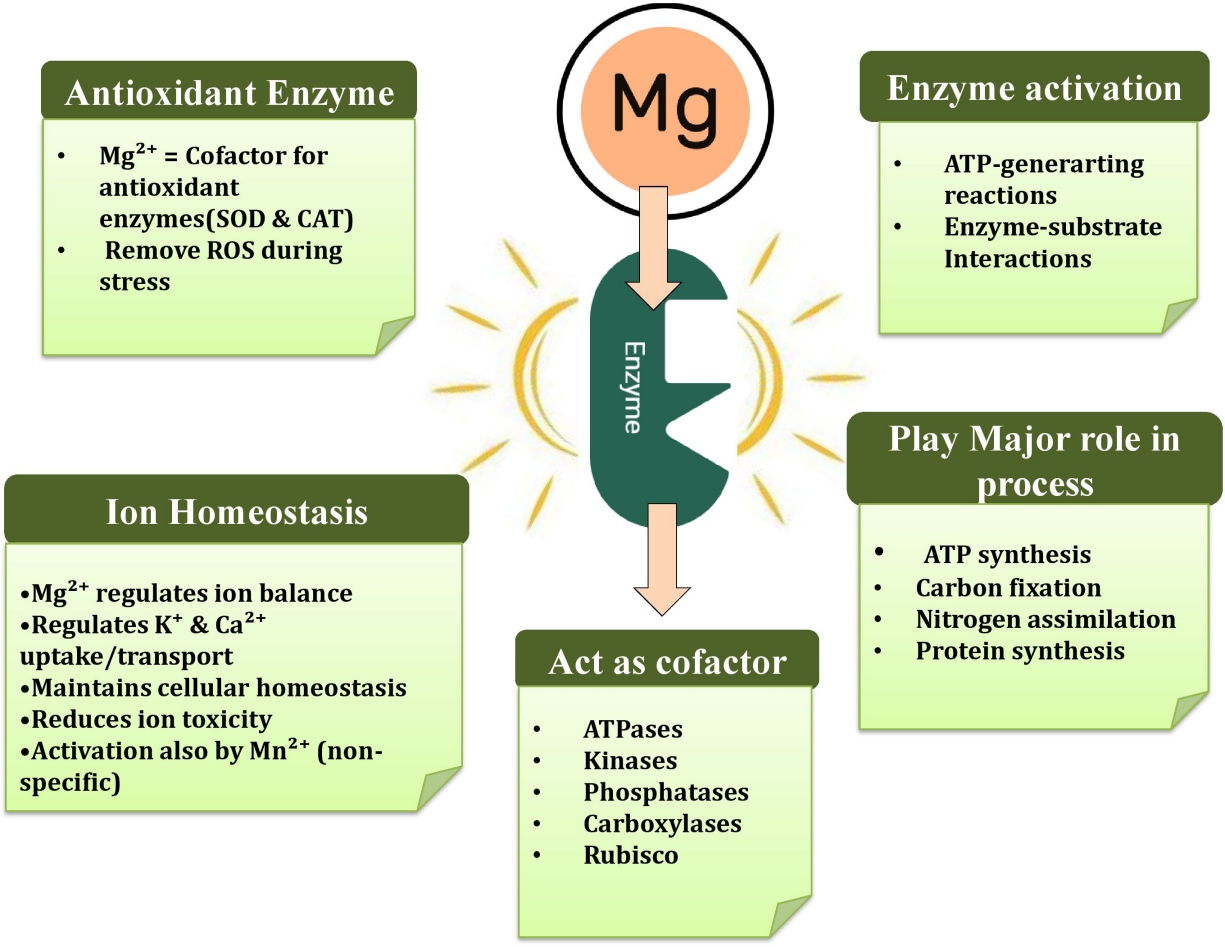
Role of Mg in maintaining cell homeostasis through the regulation of enzyme activities.

Magnesium is an essential cofactor in plant physiological processes, playing a vital role in activating a wide range of enzymes. In addition to enzyme activation, Mg facilitates enzyme-substrate interactions, induces conformational changes in enzymes, and directly participates in catalytic reactions. It is crucial for stabilizing enzymes involved in ATP production, as Mg^2+^ binds to ATP and enzyme sites to maintain structural stability and catalytic function. Furthermore, Mg stabilizes negative charges on substrates and encourages the adoption of active enzyme conformations, either as a dissociable cofactor or by binding to substrates and modifying them to improve enzyme-substrate interactions (Chaudhry et al., 2021; Ishfaq et al., 2022). The role of Mg in sustaining cellular homeostasis by regulating enzyme activities is illustrated in **Figure 1**.

Magnesium is a cofactor for over 300 enzymes, including important enzymes like ribulose-1,5-bisphosphate carboxylase/oxygenase (Rubisco), ATPases, protein kinases, phosphatases, and carboxylases (Chaudhry et al., 2021; Tian et al., 2021; Ishfaq et al., 2022). Moreover, Mg is essential for the proper functioning of enzymes such as fructose-1,6-diphosphatase, RuBP carboxylase, glutamate synthetase, and RNA polymerase, which are involved in key processes like ATP synthesis, carbon fixation, nitrogen assimilation, and protein synthesis (Pandey, 2018; Chaudhry et al., 2021).

In the case of ATPases, phosphorylases, and phosphokinases, Mg^2+^-ATP rather than free ATP serves as the substrate. The high concentration of Mg^2+^ helps maintain the high pH required for ATPases to transfer energy-rich phosphoryl groups in chloroplasts and the cytoplasm (Pilotelle-Bunner et al., 2009). Moreover, Mg acts as a bridging component between adenosine diphosphate (ADP) during ATP synthesis. In plants with sufficient Mg levels, nucleoside triphosphates primarily exist in the form of Mg complexes. Magnesium also influences ion homeostasis by regulating the uptake and transport of other essential nutrients, such as K and Ca. Maintaining this balance is crucial for proper cellular function and for reducing the toxic effects of excessive ions, such as Ca (Jahnen-Dechent and Ketteler, 2012).

Although Mg activation is not exclusive and can be achieved by other cations, such as manganese (Mn^2+^), its primary role is catalyzing the transfer of phosphate or carboxyl groups. Magnesium often works alongside other cations, mainly K^+^, to activate various enzymes, including acetic thiokinase, pyruvate kinase, and glutathione synthetase (Pandey, 2018). Additionally, Mg significantly influences plant respiratory metabolism, phosphate mutases, phosphokinases, and macronutrient absorption, thereby enhancing nutrient uptake and utilization (Kanjana, 2020; Zhang et al., 2023). Magnesium also plays an essential role in enzyme kinetics, influencing parameters such as substrate affinity, reaction rates, and enzyme regulation in vital processes such as gluconeogenesis, glycolysis, and the Krebs cycle (Pilotelle-Bunner et al., 2009).

Furthermore, Mg acts as a cofactor for antioxidant enzymes in plants, including SOD and CAT, which help remove ROS produced during stress (Uzilday et al., 2017). Low Mg levels can reduce antioxidant enzyme activity, thereby increasing oxidative stress and cell damage. However, in some cases of Mg deficiency, enzymes like CAT and POD may show increased activity (Chaudhry et al., 2021). Conversely, CAT activity may decline or remain unchanged, highlighting the complexity and sensitivity of the plant’s antioxidant system under severe deficiency (Hauer-Jákli and Tränkner, 2019).

#### 4.2.2 Impact of magnesium on plant biochemical pathways

Magnesium plays an essential role in photosynthesis as a crucial component of chl molecules that capture light energy and convert carbon dioxide into organic compounds (Tränkner and Jaghdani, 2019). In respiration, Mg participates in ATP synthesis and the metabolism of sugars and organic acids. Enzymes involved in respiration, such as pyruvate dehydrogenase and ATP synthase, require Mg for optimal activity. Inadequate Mg availability can disrupt these processes, leading to metabolic imbalances, decreased energy production, and altered carbon allocation within plants. Magnesium is also involved in carbon metabolism, including the synthesis and breakdown of carbohydrates, and enzymes such as phosphoenolpyruvate carboxylase and sucrose synthase rely on Mg for catalytic activity. Thus, disruptions caused by Mg deficiency or imbalance in these pathways can adversely affect plant growth, development, and yield (Zhang et al., 2023).

Nucleic acids are crucial for living organisms and serve various roles in plant metabolism. Magnesium is involved in stabilizing, synthesizing, and ensuring the proper functioning of nucleic acids (Sreedhara and Cowan, 2002). Ribosomes are macromolecules composed of proteins and ribonucleic acids, which are essential for protein biosynthesis. The active form of ribosomes involves the assembly of two subunits, which require Mg to facilitate their binding. Therefore, Mg deficiency also impairs protein biosynthesis (Fischer et al., 1998; Marschner, 2012).

Most (60-90%) of the Mg in optimally supplied plants can be extracted with water (Finck, 1992). The ‘metabolic Mg pool’ is mainly located in the cytoplasm and chloroplasts and is maintained by regulatory processes. These processes are specifically adapted to meet the actual metabolic needs, using the vacuole as a Mg storage compartment. The requirement for metabolic Mg is sustained through the import and export of Mg from the vacuole (Marschner, 2012). Magnesium also functions as a cation in maintaining cation-anion balance and as an osmotically active ion in cell turgor regulation (Marschner, 2012).

Finally, Mg deficiency causes oxidative stress in crop plants (Cakmak and Marschner, 1992; Cakmak et al., 1994; Candan and Tarhan, 2003; Tewari et al., 2006; Mengutay et al., 2013). The level of stress depends on the imbalance between ROS production and antioxidant enzyme activity (Keunen et al., 2013).

## 5 Magnesium as a regulatory cation

Magnesium is increasingly recognized as a regulatory cation, not just a structural or metabolic component, but one that governs a wide range of physiological and molecular processes vital for plant growth and stress adaptation. Unlike other macronutrients that primarily function as building blocks, Mg acts as a dynamic modulator of enzymatic activity, signaling pathways, and gene expression, thereby influencing cellular homeostasis under both optimal and stress conditions (Ahmed et al., 2023). Its unique ability to form complexes with ATP and other nucleotides positions Mg at the center of energy metabolism, ensuring efficient phosphorylation reactions and the activation of kinases that regulate stress-responsive signaling cascades.

Magnesium stabilizes ribosomal structures and facilitates accurate translation, both of which are vital for synthesizing stress-protective proteins during adverse environmental conditions. Beyond its metabolic roles, Mg functions as a signal integrator, affecting hormonal pathways such as abscisic acid (ABA), ethylene, and jasmonic acid, which control stomatal regulation, osmotic adjustment, and defense responses (Guo et al., 2015). Recent studies reveal that Mg availability modulates the activity of transcription factors like *WRKY*, *MYB*, and *bZIP*, thereby altering the expression of genes involved in antioxidant defense, ion transport, and osmoprotectant biosynthesis. Furthermore, Mg regulates ionic balance by interacting with Ca^2+^ and K^+^ transport systems, maintaining membrane potential and preventing cytotoxicity under salinity or drought stress.

At the cellular level, Mg deficiency triggers secondary messengers, such as ROS, which activate stress signaling networks, whereas Mg sufficiency reduces ROS accumulation by upregulating antioxidant enzymes, such as SOD and CAT (Zheltova et al., 2016). Emerging evidence also indicates that Mg plays a role in epigenetic regulation, as Mg-dependent enzymes participate in chromatin remodeling and DNA methylation, thereby influencing stress-responsive gene expression patterns. This regulatory aspect of Magnesium as a regulatory cation thus surpasses its traditional view as a passive nutrient, positioning it as a central hub in the molecular circuitry of stress adaptation. Harnessing this property through Mg fertilization, foliar sprays, or breeding strategies that enhance Mg uptake and utilization efficiency offers a promising avenue for developing climate-resilient crops (Mukherjee et al., 2025). Future research should focus on decoding Mg-sensitive signaling networks and identifying Mg-responsive cis-regulatory elements to fully exploit its regulatory potential in crop improvement programs.

## 6 Magnesium and plant abiotic stress tolerance

In the context of abiotic stress, Mg plays a vital and multifaceted role in enhancing plant tolerance. In this section, we explore how Mg protects plants under various abiotic stress conditions, emphasizing its crucial role in safeguarding plants. Additionally, we will present case studies demonstrating Mg’s role in enhancing plant resilience to stress.

**Table 1** summarizes an overview of Mg-mediated stress adaptation in various plant species.

**Table 1:** Mg-mediated stress adaptation in plants.

Crop	Stress type	Mg application	Mg source & rate	Crop response	References
Tomato (*Solanum lycopersicum*)	Mg deficiency	Soil	1 mM MgSO_4_·7H_2_O	↑ carbohydrate & carotenoid biosynthesis; ↑ amino acids; improves fruit quality	Ishfaq et al. (2025)
Rice (*Oryza sativa*)	Salt stress	Foliar	MgSO_4_ (2–5 mM)	↑ Rubisco activity; ↑ chlorophyll; ↑ Mg/Na & K/Na ratios	Liu et al. (2025)
Grapes (*Vitis vinifera*)	Nutrient deficiency	Foliar	MgSO_4_·7H_2_O at 3 mM	↑ Leaf area (37.2%); ↑ Dry weight (35.9%)	Bai et al. (2024)
Peanut (*Arachis hypogaea*)	Salt stress	Soil	MgSO_4_	Improved cation homeostasis; ↓ Na uptake	Wang et al. (2024)
Tomato	Greenhouse stress	Foliar	0.50% MgSO_4_	↑ Fruit weight; ↑ chlorophyll	Nas and Kömürkara Zengin (2024)
Soybean (*Glycine max*)	Drought stress	Soil	1.7 cmolc dm^-^³ Mg	↑ RWC; ↑ pigments	Santos et al. (2023)
Red Clover (*Trifolium pratense*)	Salinity stress	Soil	2 mM MgSO_4_	↑ Biomass; improved photosynthesis	Sharavdorj et al. (2022)
Tall Fescue (*Festuca arundinacea*)	Salinity stress	Soil	2 mM MgSO_4_	↑ Dry weight; improved forage quality	Sharavdorj et al. (2022)
Rice	Salt stress	Foliar	Mg addition	↑ Secondary metabolites; ↑ antioxidant enzymes	Xuan et al. (2022)
Arabidopsis (*Arabidopsis thaliana*)	Al stress	Soil	Mg addition	↓ NO synthesis; improved Al tolerance	Li et al. (2020b)
Himalaya ginseng *(Panax notoginseng)*	Cd stress	Soil	Mg supplementation	↓ Cd uptake by reducing NO-mediated pathways	Li et al. (2020a); He et al. (2022)
Pepper (*Capsicum annuum*)	Salt stress	Soil	Mg addition	↓ Oxidative stress; maintained growth	Zirek and Uzal (2020)
Wheat (*Triticum aestivum*)	Al stress	Foliar	Mg foliar application	↑ Malate & citrate exudation	Kibria et al. (2021)
Tomato	Mg deficiency	Hydroponic	1 mM MgSO_4_·7H_2_O	↑ Auxin biosynthesis; ↑ root size	Ishfaq et al. (2021)
Wheat	Heat stress	Soil	Adequate Mg	↑ Seed weight (24 → 41 mg)	Cakmak (2013); Mengutay et al. (2013)
Maize (*Zea mays*)	Heat stress	Soil	Mg fertilization	↑ Photosynthesis; ↓ leaf damage	Mengutay et al. (2013)
*Plantago crassifolia*	Salt stress	Soil	Mg supplementation	Restored reproductive development	Grigore et al. (2012)
Rice	Cd stress	Hydroponic	Mg addition	↓ Cd accumulation	Chou et al. (2011)
Strawberry (*Fragaria × ananassa*)	Salt stress	Soil	MgSO_4_	↑ Chlorophyll; ↑ growth	Yildirim et al. (2009)
Wheat & Radish (*Raphanus sativus*)	Zn stress	Soil	Mg supplementation	↓ Zn rhizotoxicity	Kinraide et al. (2004)
Japanese mustard spinach (*Brassica rapa*)	Cd stress	Soil	Mg addition	↓ Cd uptake; improved growth	Kashem and Kawai (2007)
*Vicia faba*/Rice bean (*Vigna umbellate*)	Al stress	Soil/hydroponic	Mg addition	↑ H^+^-ATPase activity; ↑ citrate exudation	Yang et al. (2007); Chen et al. (2015)

"↑", increase; "↓", decrease.

### 6.1 Salt stress

Mg plays a vital role in reducing the adverse effects of salt stress on plants. Salt stress, caused by increased soil salinity, markedly affects plant growth and development. Soil salinity damages plant growth and productivity mainly through two processes: osmotic stress and ionic stress, primarily due to the build-up of Na^+^ (Munns and Tester, 2008; Horie et al., 2012).

Several studies have highlighted the beneficial effects of Mg in alleviating salt stress in plants. For instance, Yildirim et al. (2009) demonstrated that exogenous Mg application to strawberry (*Fragaria × ananassa*) plants increased chl content and improved plant growth. Likewise, Grigore et al. (2012) reported that Mg supplementation restored reproductive development in salt-stressed *Plantago crassifolia*. Zirek and Uzal (2020) also found that the addition of Mg ameliorated the toxic effects of salt stress on pepper seedlings by reducing oxidative stress and maintaining optimal growth. Similarly, Liu et al. (2025) reported that foliar application of Mg enhances salt tolerance in rice (*Oryza sativa*) by improving Rubisco enzyme activity, total chl content, and Mg/Na, K/Na, P/Na, and Ca/Na ratios. Moreover, Mg supplementation notably enhances rice seedlings’ salt tolerance by increasing the synthesis of secondary metabolites and antioxidant enzymes, such as CAT, SOD, and others (Xuan et al., 2022). Wang et al. (2024) investigated the role of Mg against salt stress in peanut (*Arachis hypogaea*) seedlings. They reported that Mg improves plant growth by regulating cation homeostasis (Ca^2+^ and K^+^) by inhibiting the uptake of excess Na^+^ and limiting the loss of K^+^ and Ca^2+^.

However, supplementing Mg in salt-stressed plants reduces toxicity by decreasing the competition between Mg^2+^ and Na^+^ ions for binding sites on the root surface (Chen and Ma, 2013). This process helps maintain a healthier balance and supports the plants’ overall health.

### 6.2 Heat stress

Heat stress occurs when temperatures surpass an optimal range, disrupting biological processes and leading to reduced growth or, in extreme cases, death. It may inhibit essential functions like photosynthesis and respiration, cause wilting, and result in cellular and protein damage. Heat stress initiates a cascade of metabolic alterations in plants, significantly affecting their growth and development. Among the vital elements for plants facing heat stress, Mg plays a critical role. Studies by Kaushal et al. (2013) and Mengutay et al. (2013) have demonstrated that exogenous Mg application effectively reduces heat-stress-induced necrosis, promotes root and shoot growth in various crops, such as chickpeas (*Cicer arietinum*), maize (*Zea mays*), and wheat (*Triticum aestivum*), by minimizing oxidative stress and boosting osmolyte and antioxidant levels. This enhancement is due to improved carbohydrate distribution within plants, which helps roots access sufficient carbon sources. This, in turn, is vital for boosting respiration, especially under heat stress, and ultimately supports overall plant resilience and performance.

Magnesium plays an essential, dual role in enhancing Rubisco function, a crucial enzyme in photosynthesis. It does this by stabilizing Rubisco’s activation state and increasing the thermal stability and catalytic activity of Rubisco activase. This dual action helps prevent ROS production under heat stress, as demonstrated by Hazra et al. (2015). Furthermore, maintaining an adequate Mg supply in plant cells supports a balance between ROS generation and antioxidant levels (Da Silva et al., 2017; Tian et al., 2021). Several studies have identified Mg mitigation strategies in heat-stressed plants, particularly in preventing photooxidation. Magnesium boosts the production of antioxidant enzymes like CAT, APX, GR, and SOD. Additionally, it supports the accumulation of osmo-protectants, such as proline and glycine betaine. Siddiqui et al. (2018) and Boaretto et al. (2020) documented these effects, emphasizing Mg’s role in protecting plant cells against oxidative damage during heat stress.

### 6.3 Metal stress

Magnesium plays a vital role in shielding plants from heavy metal stress, which results from the excessive accumulation of toxic metals such as lead (Pb), cadmium (Cd), and mercury (Hg) in soil, thereby impairing plant growth and development (Saini et al., 2021). Magnesium contributes to this protection through a combination of physicochemical and physiological mechanisms. At the root surface, Mg can limit the uptake of heavy metals by competing for binding sites or by reducing electrostatic attraction to cationic metals. Internally, it supports essential biochemical processes, including maintaining membrane stability, regulating enzyme activity, and reinforcing antioxidant defense systems that mitigate oxidative damage (Rengel et al., 2015). Collectively, these functions make an adequate Mg supply crucial for reducing the negative impacts of heavy metal contamination in soils.

The mechanisms by which Mg alleviates metal stress include competitive ion uptake, stabilization of cell membranes, activation of enzymes, enhanced antioxidant activity, and the regulation of osmotic balance and nutrient homeostasis. Magnesium also influences chelation of heavy metals, modulation of stress signaling, and gene expression related to metal tolerance, such as *MGT1* (Mg transporter genes) and *WRKY* transcription factors (Tian et al., 2021). These coordinated actions further highlight Mg’s central role in protecting plants from the harmful effects of heavy metal exposure.

Several studies specifically demonstrate Mg’s protective effects against metals such as Cd, Cu, and Zn. For instance, Mg reduces Cd accumulation by decreasing nitrate reductase-mediated NO production in *Panax notoginseng* roots (Li et al., 2020a; He et al., 2022). Magnesium also decreases Cd uptake and toxicity in Japanese mustard spinach, leading to improved growth (Kashem and Kawai, 2007), and reduces Cd accumulation in rice seedlings (Chou et al., 2011). Kinraide et al. (2004) similarly reported that Mg supplementation alleviated Zn rhizotoxicity in wheat and radish by altering physiological mechanisms.

Based on these findings, Mg alleviates metal toxicity through two primary pathways: (i) physicochemical interactions at the root surface involving the cell wall and plasma membrane, and (ii) intracellular physiological protection mediated by Mg^2+^. The latter includes enhanced H^+^-ATPase activity, synthesis and exudation of organic acid anions, sequestration of metal ions within cellular compartments, and strengthening of antioxidant defenses, as reported by Janicka-Russak and Kabała (2012).

In the case of Al toxicity, numerous studies have demonstrated Mg’s beneficial effects. In *Vigna umbellata*, Mg application—either to soil or hydroponic media—reduces Al toxicity by regulating H^+^-ATPase activity, maintaining ion balance, and supporting enzyme function. It also preserves Mg and Ca concentrations in root tips (Yang et al., 2007). A well-established mechanism of Al tolerance is the Mg-dependent synthesis and exudation of organic acid anions such as malate and citrate from plant roots (Ryan et al., 2001; Silva et al., 2001; Kibria et al., 2021). Magnesium is essential for organic acid synthesis (Bose et al., 2011) and promotes H^+^-ATPase activity on the plasma membrane (Ghorbanian et al., 2019), which facilitates carboxylate exudation into the rhizosphere. The resulting formation of less toxic Al–organic acid complexes greatly enhances Al resistance (Kochian et al., 2015).

Further supporting evidence comes from Kibria et al. (2021), who showed that foliar Mg application in Al-stressed wheat stimulates exudation of malate and citrate. Li et al. (2020b) demonstrated that Mg reduces Al toxicity in *Arabidopsis* by lowering NO synthesis. Additionally, Mg increases plasma membrane H^+^-ATPase activity and citrate exudation in species such as *Vicia faba* L. and rice bean roots (Yang et al., 2007; Chen et al., 2015). Together, these findings underscore Mg’s critical role in mitigating Al stress by influencing organic acid metabolism, membrane function, and broader physiological processes.

### 6.4 Light and UV stress

Magnesium plays a crucial role in shielding plants from photooxidative damage caused by high light intensity and UV-B radiation (Mu and Han, 2024). Excess light energy can overexcite photosystems, resulting in ROS formation and photoinhibition. Adequate Mg supply stabilizes chl-protein complexes and preserves thylakoid membrane integrity, ensuring efficient energy transfer within photosystems. Magnesium improves non-photochemical quenching (NPQ), a vital photoprotective process that dissipates surplus energy as heat, thus preventing damage to photosystem II (PSII). Additionally, Mg supports the proper functioning of the oxygen-evolving complex and aids rapid repair of PSII reaction centers under stress conditions.

Under UV-B stress, Mg supplementation has been shown to upregulate antioxidant enzymes, including SOD, APX, and CAT, which collectively detoxify ROS and maintain cellular redox balance (Uzilday et al., 2017). Magnesium also promotes the accumulation of phenolic compounds and flavonoids, which act as UV-screening molecules and antioxidants, reducing oxidative damage to chloroplasts. Studies indicate that Mg deficiency exacerbates photooxidative stress by impairing chl synthesis, reducing Rubisco activity, and limiting ATP production, ultimately decreasing photosynthetic efficiency. Conversely, adequate Mg supply improves electron transport rates, enhances cyclic photophosphorylation, and maintains the proton gradient across thylakoid membranes, which is essential for ATP synthesis and photoprotection. These mechanisms underscore Mg’s critical role in safeguarding the photosynthetic machinery under excessive light and UV-B stress, contributing to plant resilience in environments with fluctuating radiation levels.

### 6.5 Nutrient imbalances

Magnesium interacts closely with other essential nutrients, including Ca, K, and phosphorus P, to maintain ionic homeostasis and metabolic balance. Magnesium acts as a counter-ion for organic acids and phosphate groups, facilitating ATP-dependent reactions and phloem loading of sucrose. However, nutrient imbalances can markedly affect Mg uptake and utilization. High concentrations of Ca or Na in the soil often antagonize Mg absorption due to competition for binding sites on root plasma membranes, leading to Mg deficiency even in Mg-rich soils (Ali et al., 2025). Similarly, excessive application of potassium fertilizers can reduce Mg uptake, resulting in physiological disorders such as interveinal chlorosis and impaired photosynthesis.

A balanced Mg supply is crucial for efficient nutrient partitioning and stress resilience. Adequate Mg enhances nitrogen assimilation by activating nitrate reductase and glutamine synthetase, while also supporting phosphorus metabolism and carbohydrate translocation. Magnesium deficiency disrupts these processes, leading to the accumulation of photoassimilates in source leaves, reduced root growth, and impaired stress tolerance. Conversely, excessive Mg can cause secondary deficiencies of Ca and K, affecting cell wall stability and stomatal regulation. Therefore, integrated nutrient management strategies that optimize Mg availability relative to other cations are essential for maintaining plant growth under abiotic stress conditions. By maintaining ionic balance and supporting enzymatic function, Mg ensures optimal physiological performance and enhances resilience against nutrient-related stress.

### 6.6 Use of magnesium fertilizers and their effects on plant stress tolerance

Several Mg fertilizers are widely used to increase soil Mg levels, thereby improving plant Mg bioavailability. There are two main types of Mg fertilizers: slowly released (e.g., Mg oxide, Mg hydroxide, dolomite, Mg carbonate, Ca-Mg phosphate, etc.) and rapidly released (Mg sulphate, Mg chloride, K-Mg sulphate, etc.) (Wang et al., 2020). A decrease in Mg content disrupts phloem loading, leading to the accumulation of photosynthetic products in the source tissue and potentially impairing photosynthetic machinery. Additionally, this situation can hinder electron transport in the electron transport chain and induce ROS production. Magnesium application helps maintain the homeostasis of photosynthate allocation between source and sink tissues and is reported to reduce membrane peroxidation levels by activating antioxidative mechanisms (Senbayram et al., 2015).

## 7 Molecular mechanisms of magnesium-mediated stress tolerance

### 7.1 Interactions of magnesium with other molecules, nutrients, and phytohormones

**Figure 2:**
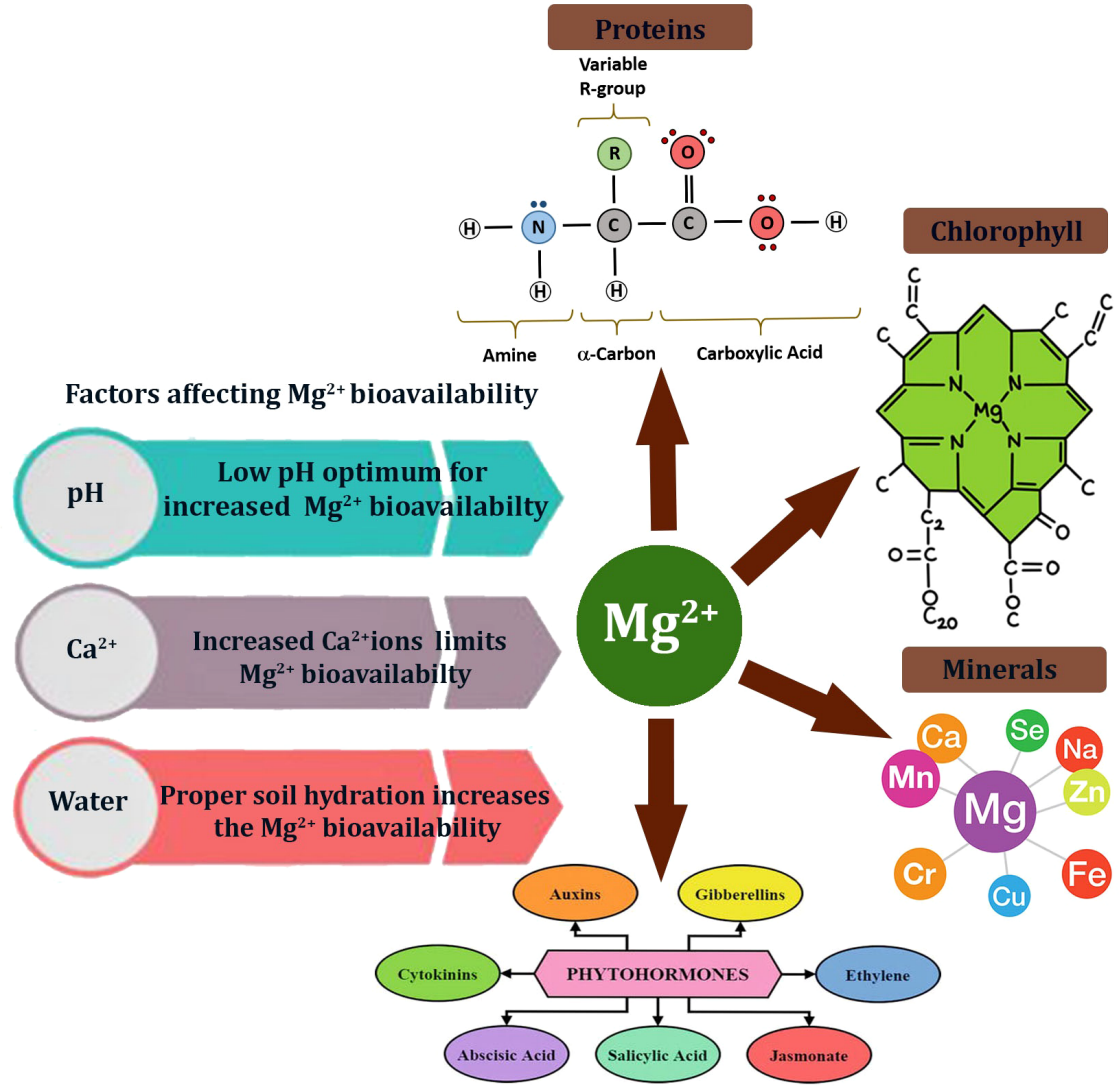
Interactions of Mg with other macro/micro molecules, including proteins, phytohormones, and nutrients.

Approximately 35% of Mg taken up is bound to chl pigments, while the remaining 65% is mainly used for protein synthesis, with many enzymes acting as cofactors (Ishfaq et al., 2022). An increased Mg supply boosts the production of secondary metabolites, such as polyphenols and catechins, thereby playing a key role in plant stress tolerance (Ruan et al., 2012). Magnesium ions are vital for activating H^+^-ATPase, which is involved in phloem loading; therefore, Mg deficiency hampers sucrose transport by inhibiting the activity of sucrose/H^+^ symporters, leading to the accumulation of photosynthates in the source tissue (Ayre, 2011; Shabala et al., 2016). Similarly, Mg^2+^ ions positively regulate photosynthesis by promoting the formation of grana through interaction with the thylakoid membrane (Puthiyaveetil et al., 2017) and by activating Rubisco. Magnesium ions are essential for Rubisco activation via binding with the carbamate group and the side chains (Glu194 and Asp193 residues) (Hazra et al., 2015). The interactions of Mg with proteins, phytohormones, and nutrients are depicted in **Figure 2**.

Magnesiums is vital for integrating multiple signaling networks, including phytohormonal regulation, ROS signaling, and calcium signaling, to coordinate plant stress adaptation. As a key cofactor for ATP and many enzymes, Mg influences hormonal biosynthesis and signaling, especially those mediated by auxin and gibberellin, which control cell elongation, root architecture, and developmental plasticity under stress. Magnesium deficiency often disrupts auxin transport by impairing PIN-FORMED (PIN) efflux carriers, resulting in altered root growth and reduced stress acclimation (Zhang et al., 2020b). Furthermore, Mg status affects gibberellin biosynthesis and signaling, as Mg-dependent enzymes regulate GA metabolism, thereby influencing growth recovery after stress.

Beyond hormonal regulation, Mg is closely connected to ROS signaling, which acts as both a stress indicator and a source of damage. Under Mg deficiency, impaired photosynthetic electron transport and ATP synthesis lead to ROS accumulation, triggering oxidative stress and activating transcription factors such as *WRKY* and *NAC* (Huang et al., 2024). Adequate Mg boosts the expression of antioxidant genes like SOD, CAT, and APX, which modulate ROS signaling to promote adaptive responses instead of cellular damage. This modulation of ROS is further linked to Ca^2+^ signaling, as Mg and Ca^2+^ interact to maintain ionic balance and membrane stability.

Stress-induced Ca^2+^ spikes serve as secondary messengers, activating calcium-dependent protein kinases (CDPKs) and calcineurin B-like proteins (CBLs) that regulate stress-responsive gene expression (Jain et al., 2018). Magnesium influences these Ca^2+^-mediated pathways by stabilizing ATP-dependent pumps and channels, ensuring controlled Ca^2+^ fluxes during stress responses. Additionally, Mg-dependent phosphorylation cascades interact with Ca^2+^ signals to modulate hormonal responses, forming a highly integrated network. Recent research indicates that Mg availability can alter cytosolic Ca^2+^ oscillations and ROS bursts, shaping the intensity and duration of stress signals perceived by the plant (Ravi et al., 2023). This cross-talk ensures that hormonal adjustments, antioxidant defenses, and ion transport function synergistically to optimize stress resilience. The connection between Mg, phytohormones, ROS, and Ca^2+^ signaling underscores Mg’s role as a master coordinator of stress adaptation rather than a passive nutrient. Understanding these interactions opens new avenues for crop enhancement, such as Mg-based fertilization regimes or genetic manipulation of Mg transporters and signaling components, to boost tolerance to drought, salinity, and temperature extremes.

The interaction of Mg with other mineral elements, such as P, positively influences the transport of photosynthetic assimilates in plants. Conversely, a higher Mg concentration reduces Ca levels, leading to Ca deficiency in plants (Puthiyaveetil et al., 2017). Similarly, cations such as Ca^2+^, NH_4_^+^, K^+^, Na^+^, and others compete with Mg for uptake from the soil and act as antagonists of Mg absorption (Wang et al., 2021; Garcia et al., 2022; Anago et al., 2023).

Magnesium ions interact with various phytohormones in plants and influence their morphogenesis. For instance, increased Mg supply, along with P, positively affects the directional growth and elongation of primary roots in *Arabidopsis* plants. This is also correlated with endogenous auxin production, as evidenced by increased expression of auxin-related genes, including *PIN2*, *PIN3*, and *AUX1* (Niu et al., 2015). Similarly, exogenous application of the hormone gibberellic acid has increased Mg accumulation in the leaves and stalks of spring wheat (Wierzbowska and Bowszys, 2008). Additionally, a transcriptomic study shows that Mg deficiency stress impairs the transportation and accumulation of carbohydrates (i.e., soluble sugars and starch) and amino acids in fruits, leading to low yield and poor quality of agricultural produce (Ishfaq et al., 2025). Moreover, Mg deficiency hampers auxin biosynthesis (*TAR/YUCs*), transport (*LAXs, PINs*), and signaling (*IAAs, ARFs*) in roots, thereby inhibiting root growth and subsequent stress resilience (Ishfaq et al., 2021).

### 7.2 Magnesium uptake and transporters

Magnesium uptake in plants is a highly regulated process that ensures an adequate supply for essential physiological functions while preventing toxicity at high levels. Plants absorb Mg mainly through root epidermal and cortical cells via Mg^2+^-permeable channels and specialized transporters. The most well-characterized transport systems belong to the *MGT/MRS2* family (Mg Transporter/Mg Responsive Sensor 2), which are part of the CorA superfamily (Maathuis and Podar, 2011). These transporters are distributed across multiple cellular compartments, including the plasma membrane, chloroplasts, mitochondria, and vacuoles, reflecting Mg’s critical role in photosynthesis, respiration, and metabolic regulation.

For example, chloroplast-localized *MGTs* assist in Mg delivery for chl synthesis and thylakoid stability, whilst mitochondrial transporters support ATP production and energy metabolism (Chen et al., 2018). The expression of *MGT* genes is highly responsive to Mg availability and environmental factors. Under Mg-deficient conditions, plants increase the expression of specific *MGT* isoforms to boost uptake and maintain cytosolic Mg homeostasis. Conversely, when Mg levels are high, transporter activity is decreased to prevent excessive accumulation, which could disrupt ionic balance and enzyme activity (Cai et al., 2012). This dynamic regulation highlights the vital role of Mg transporters in maintaining optimal Mg levels across tissues and organelles.

Magnesium status greatly influences the expression of stress-responsive genes in plants, serving as a critical factor in transcriptional reprogramming under adverse environmental conditions. Magnesium deficiency disrupts cellular balance by impairing chl synthesis, ATP production, and ribosomal stability, thereby activating stress signaling cascades (Tian et al., 2021). These disturbances induce transcription factors such as *DREB*, *WRKY*, *MYB*, and *NAC*, which regulate genes involved in osmotic adjustment, antioxidant defenses, and hormonal signaling. Conversely, Mg sufficiency sustains photosynthetic efficiency and energy metabolism, reduces stress-induced gene activation, and supports basal levels of protective gene expression (Vikanksha et al., 2025). In plants lacking sufficient Mg, these genes are often downregulated, leading to excessive ROS accumulation and oxidative damage.

Moreover, Mg availability influences hormonal crosstalk at the transcriptional level, particularly within pathways regulated by ABA and ethylene, which control stomatal closure, senescence, and stress acclimation. For instance, ABA biosynthesis genes such as *NCED* and ABA-responsive transcription factors show heightened expression under Mg deficiency, intensifying stress responses, whereas Mg sufficiency modulates ABA signaling to optimize water-use efficiency (Kaur et al., 2021). Recent transcriptomic and panomic studies suggest that Mg may also exert epigenetic control over stress-responsive genes via chromatin remodeling and DNA methylation, owing to its role as a cofactor for enzymes involved in methyl group transfer. This demonstrates that Mg is far more than a structural or metabolic component; it functions as a dynamic regulator of gene expression networks linking nutrient status with stress adaptation.

### 7.3 Sensing low magnesium in plants

Plants have developed sophisticated mechanisms to detect and respond to Mg deficiency, ensuring their survival under changing nutrient conditions. Magnesium is vital for many cellular processes, including chl synthesis, enzyme activation, and ATP-dependent reactions. When Mg availability decreases, plants detect this imbalance primarily through changes in cytosolic Mg^2+^ concentration and shifts in cellular energy status, as indicated by the ATP/ADP ratio (Kleczkowski and Igamberdiev, 2021).

A reduction in Mg disrupts ATP stability and phosphate transfer reactions, which act as early metabolic signals of deficiency. This sensing process triggers a cascade of molecular events involving CBLs and CIPKs, key components of calcium-mediated signaling networks. These proteins function as sensors and regulators, translating ionic fluctuations into adaptive responses. Under Mg-deficient conditions, CBL-CIPK complexes modulate the activity of Mg transporters, particularly members of the *MGT/MRS2* family, thereby enhancing Mg uptake and restoring homeostasis (Tian et al., 2021). This regulation ensures that Mg transport is prioritized even under competing ionic conditions, such as high Na^+^ or Ca^2+^ levels in saline soils.

In addition to signaling proteins, Mg deficiency triggers transcriptional reprogramming. The *bZIP* and *MYB* transcription factor families are activated to regulate stress-responsive genes. These transcription factors promote the expression of genes involved in ion transport, antioxidant defense, and metabolic adjustments. For instance, *bZIP* factors often regulate carbohydrate metabolism and energy signaling, while MYB proteins influence phenylpropanoid pathways, enhancing the synthesis of protective compounds such as flavonoids and phenolics (Han et al., 2023). These metabolites not only mitigate oxidative stress but also contribute to structural reinforcement under nutrient stress. Magnesium deficiency also affects hormonal signaling pathways. ABA levels typically rise under low Mg conditions, coordinating stomatal closure and osmotic adjustment to conserve water and maintain ionic balance (Liu et al., 2022). Auxin signaling is similarly modulated, influencing root architecture to improve soil exploration and Mg acquisition. These hormonal changes work in concert with nutrient signaling to optimize resource allocation during stress. At the cellular level, Mg scarcity induces ROS accumulation due to impaired photosynthetic electron transport and ATP synthesis. ROS act as secondary messengers, amplifying stress signals and activating antioxidant defense systems. This interplay between ROS and nutrient signaling ensures a rapid and integrated response to Mg deficiency (Ahmed et al., 2023).

Understanding these sensing and signaling mechanisms is vital for developing Mg-efficient crop varieties. Future research should focus on identifying Mg-specific sensors, analyzing cross-talk between Mg and other nutrient signaling pathways, and characterizing promoter regions of Mg-responsive genes. Such insights will facilitate molecular breeding and biotechnological interventions, including CRISPR-based editing of regulatory genes, to improve Mg uptake and use under stress conditions. Ultimately, enhancing Mg sensing and response will support resilient crop production systems in nutrient-limited and climate-challenged environments.

### 7.4 Regulation under stress

Abiotic stresses such as salinity, drought, and nutrient imbalances significantly affect Mg uptake and transporter activity. Stress-induced alterations in root architecture, membrane potential, and ion fluxes often hinder Mg acquisition. For instance, high Na^+^ concentrations in saline soils compete with Mg^2+^ for transport sites, thereby decreasing Mg uptake (Yang et al., 2025). Plants counteract these effects through intricate signaling networks involving Ca^2+^ sensors, calcineurin B-like proteins (CBLs), and CBL-interacting protein kinases (CIPKs), which regulate *MGT* expression and activity (Kaya et al., 2024). Furthermore, phytohormones such as ABA and auxin play vital roles in stress adaptation by regulating transporter gene expression and root development to enhance Mg acquisition under challenging conditions (Kumar et al., 2022). ROS, commonly produced during stress, also serve as signaling molecules that affect Mg transport. Increased ROS levels can induce transcriptional changes in *MGT* genes, connecting Mg uptake to oxidative stress responses (Kong et al., 2020).

Regulation of the *MRS 2/MGT* family of Mg transporters under stress is a crucial adaptive mechanism that allows plants to maintain Mg homeostasis and survive in challenging environmental conditions. Members of this family, part of the CorA-like transporter superfamily, play vital roles in Mg uptake, movement, and storage across various cellular membranes, including the plasma membrane, tonoplast, and organellar membranes such as those of chloroplasts and mitochondria (Liu et al., 2023). Under stress conditions like drought, salinity, and extreme temperatures, the demand for Mg and its distribution patterns change significantly, necessitating dynamic regulation of these transporters at both transcriptional and post-translational levels.

Transcriptomic analyses have revealed that genes encoding *MRS2/MGT* transporters are frequently upregulated in response to stress, such as Mg deficiency or osmotic stress, thereby enhancing Mg uptake and correcting ionic imbalances. For example, *Atmrs 2-1* and *Atmrs 2–4* in *Arabidopsis*, along with their homologues in rice and soybean, increase expression under salt and drought stress, supporting improved Mg absorption and maintaining photosynthetic function (Conn et al., 2011). This regulation is closely linked to signaling pathways controlled by ABA and ROS, which act as secondary messengers, activating stress-responsive transcription factors such as *WRKY, MYB*, and *bZIP*, which in turn regulate *MRS 2/MGT* gene expression.

In addition to transcriptional control, evidence indicates that these transporters undergo post-translational modifications, like phosphorylation, which may influence their activity and localization during stress. Furthermore, Mg transport via *MRS 2/MGT* channels is often coordinated with other cation transport systems, such as those for Ca^2+^ and K^+^, to sustain ionic balance and osmotic regulation during stress adaptation (Leher, 2022). Recent research employing CRISPR/Cas-based knockouts and overexpression variants has demonstrated that modifying *MRS 2/MGT* genes can significantly improve plant tolerance to abiotic stresses, highlighting their essential role in stress resilience. Additionally, panomic analyses suggest that *MRS 2/MGT* regulation integrates with metabolic adjustments, including increased ATP production and stabilizing ribosomal function, which are Mg-dependent processes crucial for stress recovery. The ability of plants to modulate *MRS 2/MGT* expression and activity in response to changing environmental conditions underscores their potential as targets for genetic improvement strategies to foster climate-resilient agriculture. Future investigations should focus on understanding the cis-regulatory elements and signaling networks that govern *MRS 2/MGT* expression under combined stress conditions, as well as their interactions with Mg-sensing mechanisms and epigenetic controls.

This regulation guarantees that Mg supply supports antioxidant enzyme activity and photosynthetic stability during stress. Recent studies indicate that Mg transporters may interact with other nutrient transport systems, such as those for potassium and calcium, to maintain ionic homeostasis under challenging environments. Understanding these regulatory mechanisms is essential for developing stress-resilient crops. Functional characterization of *MGT* isoforms, their promoter regions, and associated signaling pathways under combined stresses will provide insights into Mg homeostasis (Anwar et al., 2025). Integrating this knowledge with molecular breeding and biotechnological approaches, such as CRISPR-based editing of transporter genes, can enhance Mg efficiency and improve plant performance in stress-prone agroecosystems.

## 8 Future perspectives and research directions

Magnesium plays a vital role in plant growth and resilience, especially under abiotic stresses like drought, salinity, heat, and nutrient imbalances. However, several limitations restrict a complete understanding of its mechanisms in stress tolerance. Major gaps include the incomplete identification of Mg transporters, their regulatory pathways, and their interactions with other nutrient transport systems during various abiotic stresses. Furthermore, the context-dependent nature of Mg responses—varying across plant species, growth conditions, and stress combinations—complicates efforts to generalize its role.

The molecular and genomic mechanisms governing Mg signaling and its interactions with stress-responsive pathways, such as ROS detoxification and hormone signaling, remain only partially explored. Furthermore, the dynamics of Mg availability in soils, influenced by pH, organic matter, and ion competition, pose challenges to its effective uptake under stress. Addressing these limitations requires a multidisciplinary research approach. Proposed areas for future research include functional genomic studies of Mg transporters and signaling molecules, integration of omics technologies to uncover Mg-responsive networks, and investigation into its synergistic interactions with other nutrients and hormones.

Research on soil-Mg-plant interactions, particularly the role of microbial communities in enhancing Mg bioavailability, will also be crucial. Practical applications in agriculture include developing Mg-enriched fertilizers, tailored foliar sprays, and precision nutrient management strategies to optimize Mg use in stress-prone regions. Moreover, breeding programs should prioritize Mg efficiency by identifying phenotypic traits and molecular markers linked to its uptake and utilization under stress, and by incorporating genome-editing technologies such as CRISPR to engineer Mg-related traits.

Advancing Mg research for climate-resilient agriculture requires a multidisciplinary approach that integrates molecular, physiological, and agronomic innovations. Future studies should prioritize the functional characterization of Mg transporters and signaling networks under combined stresses such as heat, salinity, and UV exposure to understand their regulatory mechanisms. Genome-wide association studies (GWAS) and CRISPR-based genome editing offer promising avenues for improving Mg uptake efficiency and stress resilience in crops.

Additionally, integrating multi-omics platforms, including transcriptomics, metabolomics, and proteomics, will help identify Mg-responsive pathways and their interactions with other nutrient and stress signaling networks. On the agronomic front, developing Mg-smart fertilizers and precision nutrient management strategies tailored for stress-prone environments will be essential to optimize Mg availability and utilization. Exploring Mg interactions with beneficial soil microbes can further enhance bioavailability and strengthen plant tolerance to abiotic stresses. Collectively, these efforts will enable the breeding and biotechnological development of Mg-efficient, stress-resilient crop varieties, contributing to sustainable production systems and global food security under changing climatic conditions. Combining these approaches can yield stress-resilient crop varieties, sustainable farming systems, and climate-smart agricultural practices that ensure global food security amid increasing environmental challenges.

## 9 Conclusion

Magnesium is a vital macronutrient that supports essential physiological, biochemical, and molecular functions in plants, including photosynthesis, enzyme activation, nutrient transport, and hormonal regulation. Adequate Mg enhances photosynthetic efficiency, stress tolerance, and growth by supporting Rubisco activation, phloem loading, and secondary metabolite production. Magnesium interacts with other nutrients, highlighting the importance of balanced fertilization, and influences root development and overall plant plasticity. Widespread Mg deficiency negatively affects plant growth, yield, and quality, particularly under abiotic stress such as salinity, heat, and heavy metals. Sustainable approaches, including precise fertilization, biofortification, and improved nutrient management, are crucial to ensure sufficient Mg uptake. Future research should focus on understanding Mg transport and signaling, its interactions with other nutrients, and leveraging omics and advanced breeding tools to enhance Mg efficiency in crops. These strategies can support the development of stress-tolerant, climate-resilient crops and sustainable agricultural systems.
